# Dysfunction of Optineurin in Amyotrophic Lateral Sclerosis and Glaucoma

**DOI:** 10.3389/fimmu.2018.01017

**Published:** 2018-05-23

**Authors:** Reka P. Toth, Julie D. Atkin

**Affiliations:** ^1^Motor Neuron Disease Research Centre, Department of Biomedical Sciences, Macquarie University, Sydney, NSW, Australia; ^2^Department of Biochemistry, La Trobe Institute for Molecular Science, Melbourne, VIC, Australia

**Keywords:** optineurin, neuroinflammation, autophagy, vesicular trafficking, amyotrophic lateral sclerosis, glaucoma

## Abstract

Neurodegenerative disorders, including amyotrophic lateral sclerosis (ALS), frontotemporal dementia, and glaucoma, affect millions of people worldwide. ALS is caused by the loss of motor neurons in the spinal cord, brainstem, and brain, and genetic mutations are responsible for 10% of all ALS cases. Glaucoma is characterized by the loss of retinal ganglion cells and is the most common cause of irreversible blindness. Interestingly, mutations in *OPTN*, encoding optineurin, are associated with both ALS and glaucoma. Optineurin is a highly abundant protein involved in a wide range of cellular processes, including the inflammatory response, autophagy, Golgi maintenance, and vesicular transport. In this review, we summarize the role of optineurin in cellular mechanisms implicated in neurodegenerative disorders, including neuroinflammation, autophagy, and vesicular trafficking, focusing in particular on the consequences of expression of mutations associated with ALS and glaucoma. This review, therefore showcases the impact of optineurin dysfunction in ALS and glaucoma.

## Introduction

Neurodegenerative diseases are characterized by the degeneration and consequent death of neurons in specific regions of the brain and/or spinal cord. A wide range of neurodegenerative disorders manifest in humans, including amyotrophic lateral sclerosis (ALS), frontotemporal dementia (FTD), Alzheimer’s disease, Parkinson’s disease, and Huntington’s disease, that affect movement, speech, memory, and sensory or cognitive functions. Similarly, glaucoma is a primary optic neuropathy that has also been categorized as a neurodegenerative disorder (1). A variable proportion of cases of these diseases result from the presence of mutations in specific genes. One of the genes mutated in ALS, as well as in glaucoma, encodes optineurin, a ubiquitously expressed protein involved in neuroinflammation, Golgi maintenance, vesicular trafficking, and autophagy.

A common pathological hallmark of neurodegenerative diseases is the presence of misfolded proteins that aggregate into insoluble intra- or extracellular inclusions. Extensive research has been undertaken to determine whether these inclusions contribute to the development of disease, but it remains controversial whether the inclusions are either harmful, by directly inducing toxicity or beneficial, by sequestering misfolded, toxic proteins. Similarly, the etiology of these conditions remains unclear. Optineurin is present within the inclusions in several neurodegenerative disorders, including ALS (2), FTD (3), Alzheimer’s disease (4), and Huntington’s disease (5). However, the role of optineurin in the pathogenesis of ALS and other diseases is not well understood. In this review, we summarize the current understanding of the role of optineurin in ALS, glaucoma, and also other neurodegenerative diseases, focusing on autophagy, vesicular trafficking, and neuroinflammation.

## ALS and FTD

Amyotrophic lateral sclerosis, also known as motor neuron disease or Lou Gehrig’s disease, is a mainly adult-onset, rapidly progressing neurodegenerative disorder. ALS is associated with the loss of upper motor neurons in the motor cortex and brainstem and lower motor neurons in the ventral horn of the spinal cord. The median period of survival following diagnosis is approximately 3 years (6), although 10% of patients live up to 10 years after symptom onset (7). Over the course of ALS, patients gradually lose the ability to walk, move, swallow, or speak, and eventually to breath. The worldwide incidence of ALS is 1.75 in 100,000 people and it is slightly higher in men than women (8).

Most cases of ALS (90%) arise sporadically, with no previous family history. However, approximately 10% of cases are familial (fALS), resulting from the presence of one or more genetic mutations. It was previously thought that sporadic ALS (sALS) has no genetic component; however, recent studies have revealed that genetic mutations may be the underlying cause of a significant proportion of sALS cases (9–11). However, studies of identical twins have also implicated both environmental and epigenetic factors in the development of ALS (12–16).

**Figure 1:**
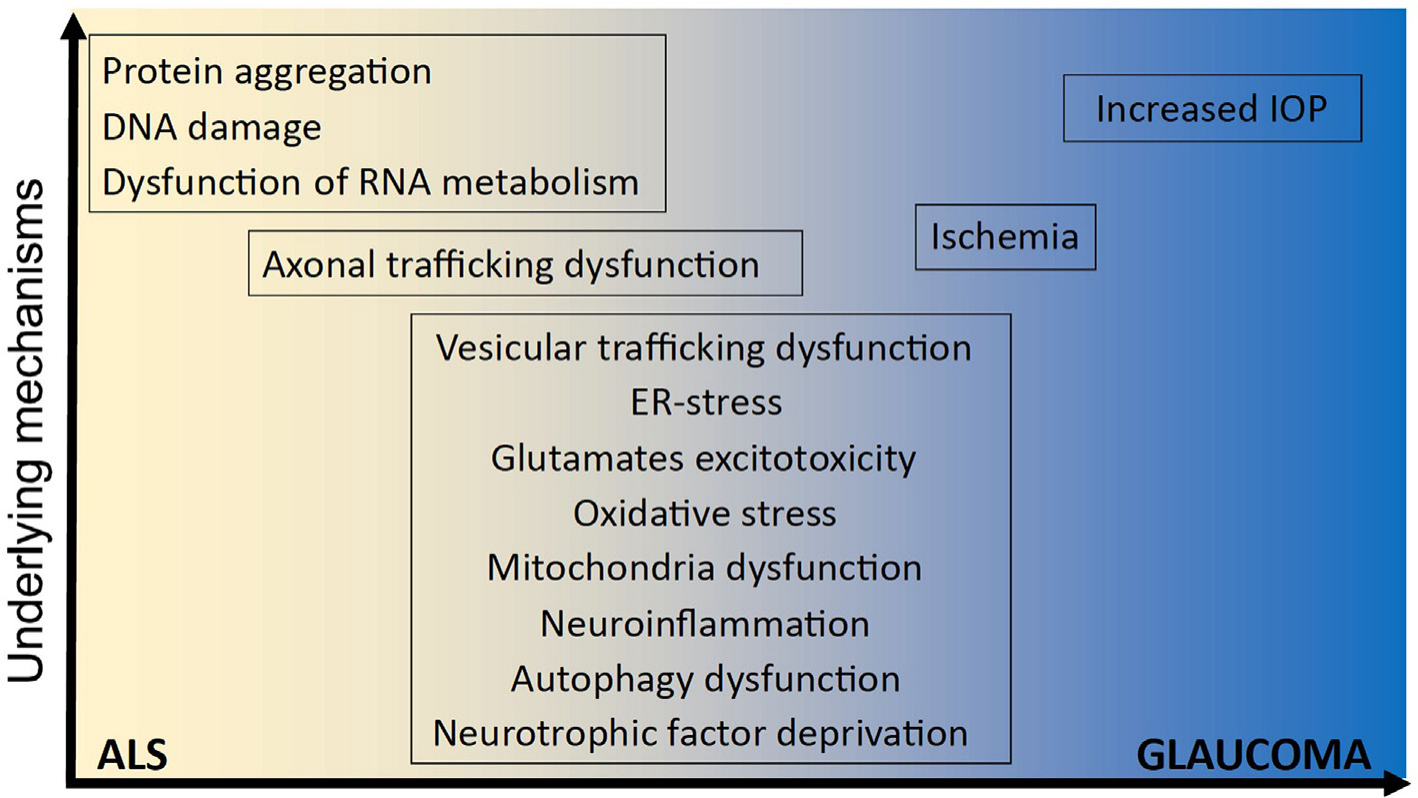
Underlying mechanisms implicated in ALS and glaucoma. It remains unclear how optineurin mutations cause amyotrophic lateral sclerosis (ALS) and glaucoma and whether neurodegeneration involves mechanisms that are unique to one disorder or common to both. However, several shared cellular mechanisms have been implicated in both disorders, namely vesicular trafficking, endoplasmic reticulum (ER) stress, excitotoxicity, oxidative stress, mitochondria dysfunction, neuroinflammation, autophagy dysfunction, neurotrophic factor deprivation, axonal trafficking dysfunction, and ischemia. In contrast, other cellular processes have been implicated in either ALS; dysregulation of RNA metabolism, DNA damage, and protein aggregation; or glaucoma; increased intraocular pressure (IOP), and IOP; but not both.

Due to improvements in next-generation sequencing methods, extensive advances have been made in the last 20 years in the underlying genetic causes of fALS. The first gene linked to ALS was superoxide dismutase-1 (SOD1) (17), and since then, more than 30 genes have been associated (18), either strongly or weakly, with ALS. Mutations in the *Chromosome 9 open reading frame 72* (*C9orf72*) gene are responsible for most fALS cases (40%) in Caucasian populations (19), followed by mutations in genes encoding SOD1 (12%), TAR-DNA-binding protein of 43 kDa (TDP-43, 4%), and fused in sarcoma (FUS, 4%) (19). Rarer cases of ALS are caused by mutations in the genes encoding TANK-binding kinase-1 (TBK-1) (20), valosin containing protein (VCP) (21), ubiquilin-2 (22), cyclin-F (23), and optineurin (2), as well as several others (Table 1). Optineurin mutations have been identified in both fALS and sALS patients, and their frequency depends on ethnicity. In Asian populations, mutations in *OPTN* account for 4% of fALS and 0.4% of sALS cases, whereas this frequency is much lower in European populations (1.5 and 0.3%, respectively) (24). Dysfunction in several cellular mechanisms has been linked with the pathophysiology of ALS (and/or glaucoma), including excitotoxicity, deregulation of RNA metabolism, endoplasmic reticulum (ER) stress, dysfunction of mitochondria, autophagy, neuroinflammation, cytoskeletal defects, and altered axonal transport (Figure 1).

**Table 1:** Genes associated with familial ALS.

Gene	Protein	Year of discovery	Reference
*SOD1*	Superoxide dismutase-1	1993	(17)
*NEFH*	Neurofilament heavy polypeptide	1994	(162)
*SETX*	Senataxin	1998	(163)
*ALS2*	Alsin	2001	(164)
*DCTN1*	Dynactin 1	2004	(165)
*VAPB*	Vesicle-associated membrane protein-associated protein B/C	2004	(166)
*ANG*	Angiogenin	2004	(167)
*CHMP2B*	Charged multivesicular body protein 2B	2006	(168)
*TARDBP*	TAR-DNA-binding protein 43 kDa	2006	(169)
*FUS*	Fused in sarcoma	2009	(170)
*ELP3*	Elongator protein 3	2009	(171)
*FIG 4*	FIG 4 phosphoinositide 5-phosphatase	2009	(172)
*C9ORF72*	Chromosome 9 open reading frame 72	2011	(173)
*SQSTM1*	Sequestosome 1	2011	(174)
*UBQLN2*	Ubiquilin-2	2011	(22)
*TAF15*	TATA-binding protein-associated factor 2 N	2011	(175)
*VCP*	Valosin containing protein	2010	(21)
*OPTN*	Optineurin	2010	(2)
*ATXN2*	Ataxin 2	2010	(176)
*SPG11*	Spatacsin	2012	(177)
*PFN1*	Profilin 1	2012	(178)
*EWSR1*	EWS RNA-binding protein 1	2012	(179)
*HNRNPA1*	Heterogeneous nuclear ribonucleoprotein 1 A	2013	(180)
*SS18L1*	nBAF chromatin remodeling complex subunit	2013	(9)
*ERBB4*	erb-b2 receptor tyrosine kinase 4	2013	(181)
*CHCHD10*	Coiled-coil-helix-coiled-coil-helix domain containing 10	2014	(182)
*MATR3*	Matrin 3	2014	(183)
*TUBA4A*	Tubulin alpha 4a	2014	(184)
*TBK-1*	Tank-binding kinase-1	2015	(20)
*GLE1*	Gle1	2015	(185)
*C21ORF2*	Chromosome 21 open reading frame 2	2016	(186)
*NEK1*	NIMA-related kinase-1	2016	(187)
*CCNF*	Cyclin-F	2016	(23)
*SFPQ*	Splicing factor proline and glutamine rich	2017	(188)
*KIF5a*	Kinesin family member 5A	2018	(189)

*In the last two decades, genome sequencing of fALS patients has identified mutations across a wide range of genes*.

In recent years, it has become increasingly recognized that ALS overlaps genetically and pathologically with FTD. Moreover, it is now an accepted dogma that ALS and FTD represent two ends of the same disease spectrum (25). FTD is the second most common form of dementia after Alzheimer’s disease, with a prevalence estimated between 10 and 20 per 100,000 individuals (26, 27). FTD is a complex disorder associated with progressive atrophy of both the frontal and temporal lobes of the brain. Clinical symptoms include behavioral changes and difficulties in speech (27, 28) [for recent reviews, see Ref. (26, 29)]. Up to 50% of ALS patients will develop FTD-like symptoms (29, 30), and approximately 15% of patients will receive a definite diagnosis of FTD. Similarly, up to half of FTD patients will experience motor neuron dysfunction, and eventually 15% will be diagnosed with ALS. Genetic mutations are present in 10–20% of FTD cases and mutations in *C9orf72, TARDBP, FUS, VCP*, or *SQSTM1*, and *TBK-1* are also associated with FTD as well as ALS. Although the role of *OPTN* mutations in FTD is controversial, they are implicated in >1% of patients (31).

## Glaucoma

Glaucoma describes a group of disorders characterized by the death of retinal ganglion cells (RGCs) and degeneration of the neural axons that form the optic nerve fibre (32). It is the second leading cause of irreversible blindness, affecting over 57 million people worldwide (33). Primary open-angle glaucoma (POAG) is the most common type of glaucoma, where disease develops despite anatomically correct ocular structures. Increased intraocular pressure is a major risk factor for glaucoma (32). Normal tension glaucoma (NTG) is a subtype of POAG, where glaucoma arises in the absence of increased intraocular pressure (34). Mutations in the gene encoding myocilin are causative in 8–36% of juvenile open-angle glaucoma cases and 2–4% of adult-onset POAG cases (34). Missense mutations in *OPTN* (35) and copy number variations of *TBK-1* (36) account for approximately 2–3% of NTG.

## Optineurin Mutations in ALS, FTD, and Glaucoma

*OPTN* was first identified as an ALS causative gene in 2010, and since then, more than 20 mutations have been described. Sequencing of six Japanese individuals with consanguineous marriages first identified three mutations: homozygous deletion of exon 5, a homozygous mutation that results in a truncated protein (Q398X), and a heterozygous missense mutation (E478G) (2). Additional ALS mutations in *OPTN* were subsequently identified in ALS patients: R96L (37), Q165X (38), A136V (39), K395K (39), I451T (39), E516Q (39), and Q454E (38) (a full list of ALS-associated mutations in *OPTN* is detailed in Table 2). However, the involvement of optineurin in FTD is still under debate. One study identified *OPTN* variants in 4.8% of FTD patients without motor neuron involvement (31), but another study examining 371 FTD cases could not detect the presence of optineurin mutations (40). In contrast, optineurin mutations have been frequently reported in glaucoma. Analysis of 54 families with autosomal dominantly inherited adult-onset POAG revealed that 16.7% of patients possessed *OPTN* mutations (35). These mutations include E50K (35), M98K (35), H486R (35), R545Q (35), H26D (41), E322K (42), E103D (43), and V148V (43) [for a detailed recent review discussing *OPTN* mutations and glaucoma, see Ref. (33)].

**Table 2:** ALS-associated mutations in *OPTN*.

Mutation	Reference
p.E478G	Maruyama et al. (2)
p.Q398X	Maruyama et al. (2)
del. Exon5	Maruyama et al. (2)
p.K95N	Belzil et al. (190)
c.1242+1G>A_insA	Belzil et al. (190)
p.T282P	Del Bo et al. (191)
p.Q314L	Del Bo et al. (191)
p.K557T	Del Bo et al. (191)
p.G23X	Del Bo et al. (191)
c.552+1delG	Del Bo et al. (191)
c.1410+4A→G	Del Bo et al. (191)
c.691_692insAG	Millecamps et al. (37)
p.R96L	Millecamps et al. (37)
p.V161M	Naruse et al. (192)
p.Q165X	Tumer et al. (193) and van Blitterswijk et al. (38)
p.Q454E	van Blitterswijk et al. (38)
c.688delG	Beeldman et al. (194)
c.493C>T	Beeldman et al. (194)
p.A136V	Li et al. (39)
p.K395R	Li et al. (39)
p.I451T	Li et al. (39)
p.E516Q	Li et al. (39)
p.E322K	Bury et al. (195)
p.V295F	Fifita et al. (24)
p.A93P	Iida et al. (196)
p.R271C	Iida et al. (196)

## Functions of Optineurin

**Figure 2:**
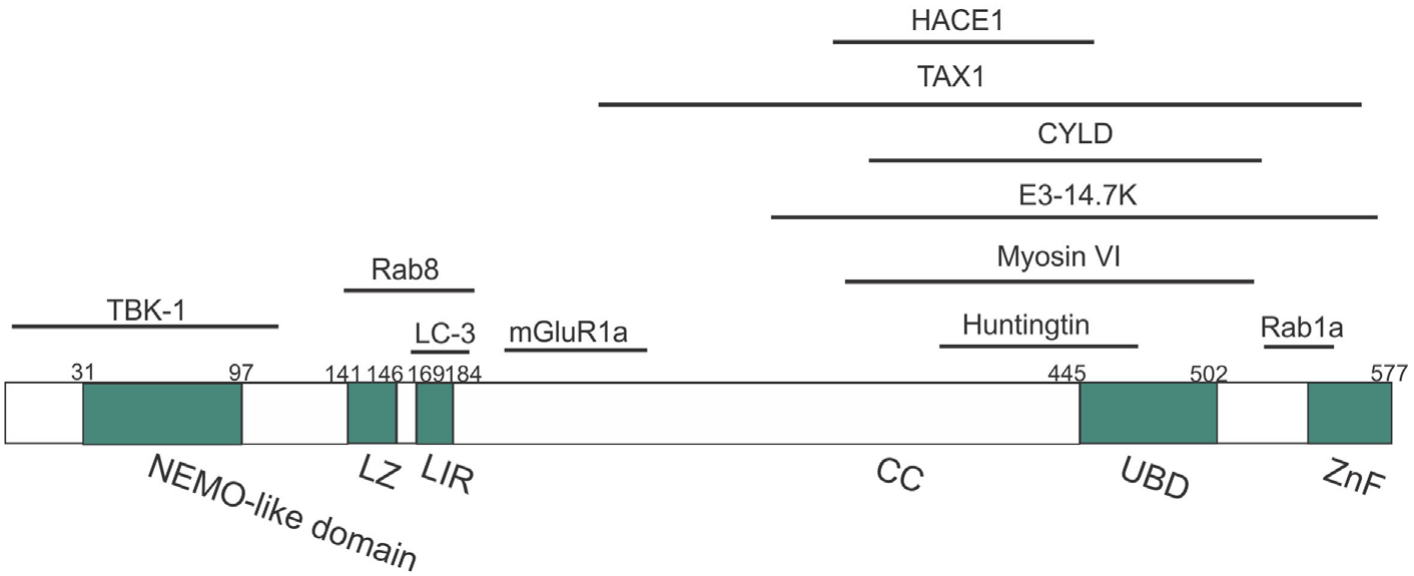
Domain structure of optineurin, illustrating possible regions that associate with its binding partners. Optineurin consists of a NEMO-like domain, a LC3-interacting (LIR) domain, a LZ domain, an ubiquitin-binding domain (UBD), coil-coiled (CC) domains, and a zinc-finger-like (ZnF) domain. TBK-1, Rab8, LC3, and mGlutR1a bind to the N-terminus whilst HACE1, TAX1, E3-14.7K, myosin VI, huntingtin, and Rab1a bind toward the C-terminus.

Optineurin is a highly conserved, 64 kDa hexameric protein, consisting of 577 amino acids (aa) (44). The putative domain structure of optineurin is illustrated in Figure 2. Optineurin is ubiquitously expressed in all human organs including heart, brain, liver, kidney, placenta, and pancreas, with exceptionally high expression in skeletal muscle (45). Optineurin normally undergoes post-translational modifications that impact significantly on its function, including phosphorylation and ubiquitination (44). One major kinase that is capable of phosphorylating optineurin on at least nine serine and two threonine residues is TBK-1 (46). Optineurin is ubiquitinated by either HECT domain and ankyrin repeat containing E3 ubiquitin protein ligase-1 (HACE1) (47) or ERAD-associated E3 ubiquitin protein ligase-1 (Hrd1) (48).

Optineurin is a multifunctional protein involved in several cellular processes. Importantly, it is an autophagy receptor (or adaptor) that is implicated in several forms of selective autophagy, including xenophagy (49), mitophagy (50), and aggrephagy (51). It also functions in vesicular trafficking (52), Golgi maintenance (53), secretion (54), and receptor recycling (55, 56). Furthermore, optineurin influences the innate immune response by negatively regulating the nuclear factor kappa B (NF-κB) pathway (45). This diversity in the functions of optineurin may originate from its numerous possible interacting partners (more than 20, Table 3; Figure 2).

**Table 3:** Comprehensive list of putative optineurin-binding partners.

Interacting partner	Binding site	Implicated cellular processes	Reference
TBK-1	1–127 amino acid (aa)	Autophagy/inflammation	(82)
Rab8	141–209 aa	Trafficking	(60)
Huntingtin	411–461 aa	Trafficking	(60)
CYLD	424–509 aa	Inflammation	(63)
mGluR1a	202–246 aa	Excitotoxicity	(144)
LC3	169–209 aa	Autophagy	(49)
Myosin VI	412–520 aa	Trafficking	(53)
TAX 1	278–577 aa	Inflammation	(86)
E3-14.7K	346–577 aa	Inflammation	(197)
HACE1	411–456 aa	Autophagy	(47)
Rab1a	532–557 aa	Autophagy	(58)
IRAK1	UN	Inflammation	(61)
Hrd1	UN	Degradation	(48)
RIPK1	UN	Inflammation	(62)
Rab12	UN	Autophagy	(136)
ATG5–ATG12 complex	UN	Autophagy	(59)
Wipi2	UN	Autophagy	(59)
Tom1	UN	Autophagy	(198)
TAXBP1	UN	Autophagy	(86)

*Known binding sites are indicated*.

*UN, unknown*.

Optineurin was first discovered at the nuclear membrane as a binding partner for adenoviral protein E3-14.7K in a yeast-two hybrid system, and it was originally named “FIP2” (45). In this study, optineurin was found to suppress the protective effect of E3-14.7K against tumor necrosis factor alpha (TNFα)-induced cell death in HEK 293 cells. Optineurin was later found to also interact with several proteins involved in autophagy, including microtubule-associated proteins 1A/1B light chain 3B (LC3) (49), p62 (57), TBK-1 (49), Rab1a (58), and the ATG12-ATG5-ATG16L1 complex (59). During protein trafficking, optineurin forms a complex with myosin VI (53) and Rab8 (60) and it is also an interacting partner of huntingtin (htt) protein (60). In addition, during the inflammatory response, optineurin interacts with interleukin-1 receptor-associated kinase-1 (IRAK1) (61), receptor-interacting protein kinase-1 (RIPK-1) (62), and ubiquitin carboxylterminal hydrolase (CYLD) (63).

## The Role of Optineurin in Neuroinflammation

### Neuroinflammation

Neuroinflammation refers to chronic or acute central nervous system (CNS)-specific, inflammatory responses. It is a complex response mediated by activated microglia and/or astrocytes and is characterized by overproduction of inflammatory cytokines that may involve T-cell infiltration into the CNS. Whilst neuroinflammation is a normal response to CNS injury, it is increasingly accepted that neuroinflammatory processes are closely linked with neurodegenerative disorders, which are age-related conditions. TNFα and interleukin-1β (IL-1β) production can result from either normal aging or the neurodegenerative processes (64). This disturbed immune system in turn may exacerbate a favorable environment for neurodegeneration.

Microglia (65) are the innate immune cells of the CNS whose primary function is to monitor the environment to detect injury and infection. Depending on the signal and surrounding milieu, microglia may acquire different phenotypes. A simplified model describes two main phenotypes (66–69): M1 or “classically activated” microglia and M2 or “alternatively activated” microglia (69). M1 microglia produce pro-inflammatory cytokines including TNFα, IL-1β, IL-6, and IL-12, reactive oxygen species (ROS), and nitrogen-monoxide. This may lead to activation of CD8+ killer T cells, which aim to eradicate the infection. In contrast, M2 microglia produce anti-inflammatory cytokines including IL-4, IL-10, and insulin-growth factor-1 (IGF-1), which consequently activate CD4+ helper T cells. M2 microglia display enhanced phagocytosis compared to M1 microglia (70). Hence, depending on the phenotypes following activation, microglia can produce either cytotoxic or neuroprotective effects and the balance between M1 and M2 is thought to be essential for a healthy CNS.

Astrocytes (71) are specialized glial cells that are highly abundant in the CNS and they possess a wide range of essential functions. Astrocytes are also key players in neuroinflammation (72), and their response may also be either beneficial or harmful. During insults, their number and cellular volume can increase abnormally in a process known as ‘reactive astrogliosis’. Astrocytes also produce pro-inflammatory cytokines in response to inflammatory stimulators and they can also be activated via molecules secreted by activated microglia. Hence, during neuroinflammation, astrocytes can be exposed to a wide variety of stimuli, leading to a complex network of intracellular events (73–75).

**Figure 3:**
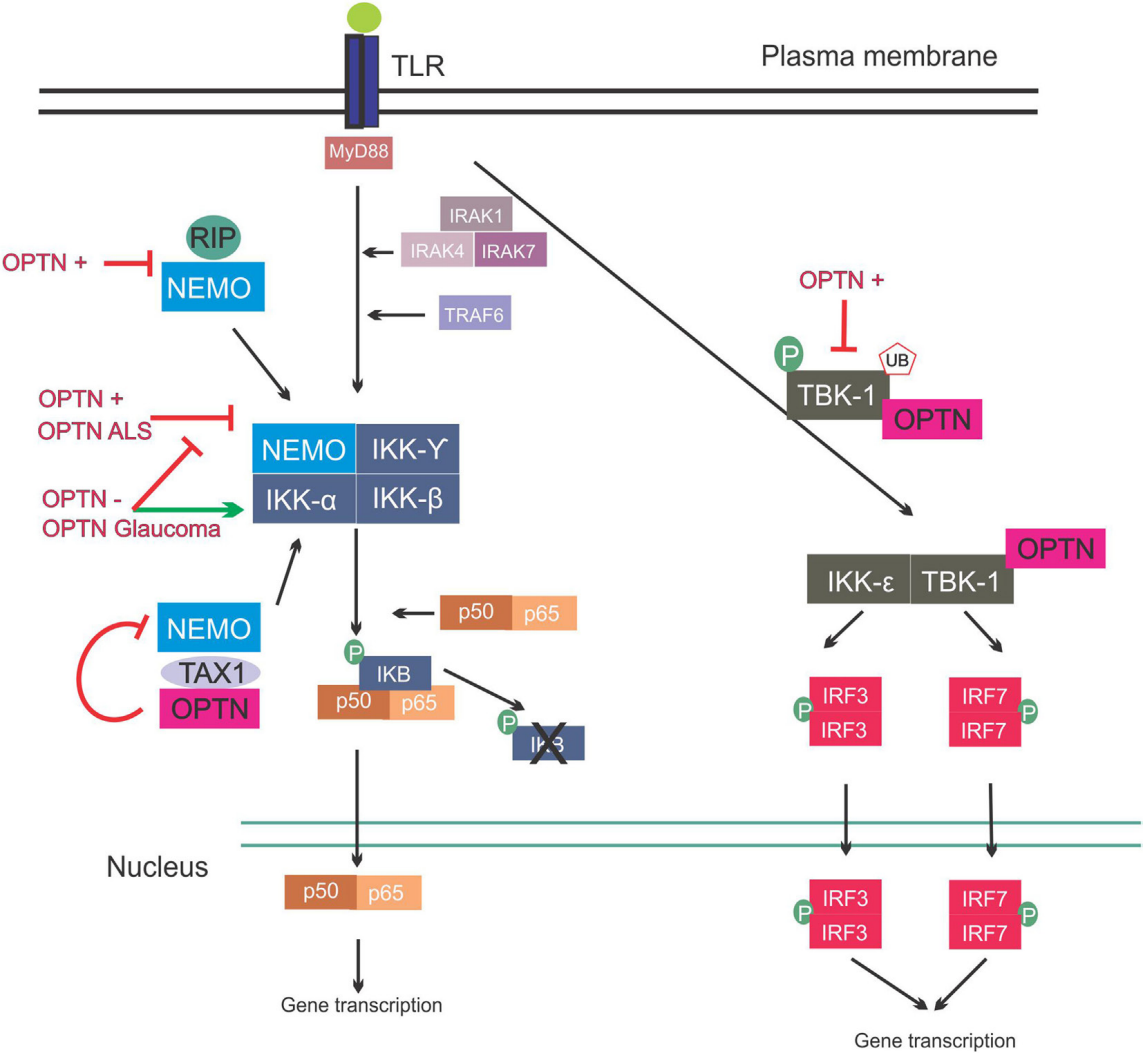
Involvement of optineurin in nuclear factor kappa B (NF-κB) signaling. Upon Toll-like receptor (TLR) signaling the NF-κB pathway is activated, leading to expression of inflammatory cytokines. This is mediated via interleukin-1 receptor-associated kinases (IRAKs) and TRAF. NEMO binds to the I kappa B (IKB) complex, leading to its phosphorylation and consequent degradation, and translocation of p50/p65 to the nucleus. NEMO also interacts with receptor-interacting protein (RIP) and trans-activating transcriptional regulatory protein of HTLV-1 (TAX1). Overexpression of wild-type or ALS mutant optineurin blocks NF-κB activation. Depletion of optineurin or overexpression of the glaucoma mutants leads to failure to inhibit NF-κB, leading to activation of this pathway. Optineurin binds to TAX1, blocking its interaction with NEMO and further inhibiting the pathway. Interferon regulatory factors are activated during TLR activation. IRF3 and IRF7 activation is mediated by TBK-1, and TBK-1 undergoes phosphorylation and ubiquitination during its activation. Optineurin binds to TBK-1, targeting it to CYLD, which inhibits its enzymatic activity. Abbreviations: OPTN−: optineurin depletion, OPTN+: optineurin overexpression, OPTN ALS: ALS mutant optineurin, OPTN Glaucoma: glaucoma mutant optineurin.

The NF-κB family of transcription factors are key regulators of cytokine production, and they provide a mechanism to respond to a wide variety of stimuli associated with inflammation. NF-κB is ubiquitously expressed in mammalian cells, including neurons, and it can protect neurons against injury and regulate neuroinflammatory reactions, as well as contribute to neuronal degeneration (76–78). The family has the following five members: NF-κB1, NF-κB2, RelA, RelB, and c-Rel. NF-κB proteins bind to kB sites in the promoters of genes encoding inflammatory cytokines, thus inducing transcription (79). In basal conditions, NF-κB dimerizes in the cytoplasm and binds to the I kappa B (IKB) complex, consisting of IKB kinase α (IKKα), IKKβ, and NF-κB essential modulator (NEMO) kinases. Upon activation, the regulatory domain of IKB undergoes phosphorylation followed by ubiquitination, and this ultimately leads to degradation of the IKB complex. This then releases NF-κB, which translocates to the nucleus and promotes gene expression (79, 80) (Figure 3).

### Optineurin’s Controversial Involvement in the NF-κB Pathway

It was initially hypothesized that optineurin would influence the NF-κB pathway because of its 53% aa similarity to NEMO (81). However, despite this similarity, optineurin did not bind to IKKβ, IKKα, or NEMO and it also lacked kinase activity (81). However, two other kinases were subsequently identified that associated with optineurin (81). Whilst these kinases were uncharacterized at the time, TBK-1 was later identified as a binding partner of optineurin (82). The first 127 aa of optineurin contain the TBK-1-binding site, and interestingly, the region 78–121 aa displays prominent homology to the TBK-1-binding site of TANK, another substrate of TBK-1 (Figure 2) (49). TBK-1 has a molecular weight of 84 kDa, similar to the unknown kinase, and it also belongs to the IKK-related family of kinases. Furthermore, as optineurin and TBK-1 are involved in immune responses, it is probable that this kinase was TBK-1. There has previously been controversy as to whether optineurin plays a role in NF-κB signaling. Whilst earlier studies failed to demonstrate a relationship between optineurin and NF-κB, more recent studies have provided evidence that optineurin negatively regulates this pathway. Depletion of optineurin abrogated the protective effect of E3-14.7K against receptor-interacting protein (RIP) and TNF receptor 1 (TNFR1)-induced death of HeLa cells (45). In contrast, depletion of optineurin did not influence p65 phosphorylation, pro-inflammatory IL-8 secretion, or the protective effect of E3-14.7K in H1299 cells, implying that optineurin was not required for NF-κB activation (83). In addition, optineurin did not associate with either NEMO or IKKβ and did not affect NF-κB signaling in murine pre-B-lymphocyte cell lines (81).

In contrast, other studies have concluded that optineurin is indeed involved in the NF-κB pathway. NEMO binds K63 poly-ubiquitinated RIP, which binds to TNFR1 during TNFα induced-NF-κB activation, and optineurin possesses a similar K63 ubiquitin-binding region to NEMO (Figure 3). This region competitively antagonized NEMO’s binding to RIP in HeLa cells, leading to decreased NF-κB activation (84). Similarly, in another study, optineurin was identified as a poly-ubiquitin-binding protein that regulates NF-κB activity (85). Another possible level of regulation of NF-κB by optineurin is through ubiquitin-binding domain (UBD)-dependent binding to trans-activating transcriptional regulatory protein of HTLV-1 (TAX1), which when ubiquitinated binds to NEMO, leading to NF-κB activation. This is mediated by binding of optineurin to TAX1-binding protein (TAXBP1), which regulates the ubiquitination of TAX1 (86) (Figure 3). Further homology with NEMO can be found in the zinc-finger domain of optineurin (87). The deubiquitinase CYLD also interacts with optineurin and is a negative regulator of NF-κB signaling through binding of RIP, similar to optineurin (88).

Despite the controversial involvement of optineurin in regulation of the NF-κB pathway, expression of optineurin itself is modulated through this pathway. The levels of optineurin increase upon treatment with either TNFα (45) or IFN (45, 81). Furthermore, the human optineurin promoter is activated by TNFα, and this is abolished by mutations in the putative NF-κB-binding site. Furthermore, overexpression of the NF-κB subunit p65 activates the optineurin promoter, providing evidence that optineurin expression is controlled by the NF-κB pathway (89).

Whilst not the focus of this review, it is important to note that optineurin is involved in antiviral and antibacterial immune responses outside of the CNS. Overexpression of optineurin inhibited IL-1β-, IRAK1-, and lipopolysaccharide (LPS)-induced NF-κB activation, whilst optineurin deficiency increased NF-κB activation in response to LPS (61). Optineurin has also been implicated in mediating the immune response to *Salmonella* infection (90). Interestingly, optineurin function in inflammation is highly dependent on its interaction with TBK-1. Pathogen associated molecular patterns (PAMPs) lead to the phosphorylation and ubiquitination of TBK-1, consequently leading to IFNβ production (91). TBK-1-mediated IRF3 activation is dependent on optineurin (92). Furthermore, optineurin was shown to dampen the antiviral innate immune response by targeting CYLD to TBK-1 to inhibit its enzymatic activity (93). Similarly, in mice expressing N-terminal truncated optineurin that lacks the TBK-1 interacting domain, PAMP activation led to decreased TBK-1 activity and low IFNβ production (91). Taken together, these findings indicate that the presence of functional optineurin is crucial for the normal innate immune response and dysfunctional optineurin can unbalance this response.

Based on the available evidence, it is likely that optineurin does indeed influence NF-κB activity, although this may happen in a cell type and stimulus-dependent manner. Furthermore, optineurin interacts with other proteins involved in NF-κB signaling including RIPK-1, which could affect its ubiquitination status via CYLD. Hence optineurin may influence the NF-κB signaling pathway by an indirect mechanism.

### Role of Optineurin in Necroptosis

Necroptosis is a programed form of necrosis linked to inflammation (94). In contrast to apoptosis, necroptosis is caspase-independent and can be triggered by TNF treatment. Activation of TNFR1 by binding of TNF leads to either inflammation via activation of the NF-κB pathway or cell death, and RIPK-1 mediates this cellular response and dictates which pathway is activated (94). In most cells, TNFR1-bound TNF leads to activation of NF-κB signaling and the production of inflammatory cytokines (95). However, upon destabilization of the RIPK-1–TNFR1–TRADD complex, cell death is triggered. K63-mediated ubiquitination of RIPK-1 promotes cell survival, but upon de-ubiquitylation, the default pro-survival function is lost. Up-regulation of RIPK-1, RIPK-3, and mixed lineage kinase domain-like protein (MLKL) can trigger necroptosis (94).

A recent study identified a major role for optineurin in RIPK-1-mediated necroptosis (62). In optineurin-depleted mice, RIPK-1, RIPK-3, and MLK levels were increased in the spinal cord and increased cell death upon TNF treatment was observed, implying induction of necroptosis. Furthermore, ubiquitination and proteasomal turnover of RIPK-1 was decreased in these animals, implying that optineurin may influence the cellular sensitivity to necroptosis by this mechanism (62). The optineurin-depleted animals also displayed degeneration and swelling of motor neuronal axons, indicating a vital role for optineurin in axonal degeneration and neuroinflammation.

## The Role of Optineurin in Vesicular Trafficking

Secretory proteins are first synthesized and folded within the ER, from where they bud via ER exit sites in membrane-bound vesicles that move towards the Golgi apparatus (96). The Rab family of small GTPases regulate all intracellular trafficking events, including recruitment of coatomer coat proteins that form the vesicular coat, vesicle budding, transport, docking, and fusion (96). The ER-derived vesicles arrive at the cis-Golgi and then transit to the trans-Golgi network (TGN), which sorts and packages proteins according to their destination: the plasma membrane, endosomes, or extracellular space (96). Motor proteins carry the transport vesicles along either actin filaments or microtubules to their destination, where they fuse with the donor membrane to release their cargo (96). Motor neurons are very long and vesicular trafficking could therefore have more severe consequences in these cells than in other types.

**Figure 4:**
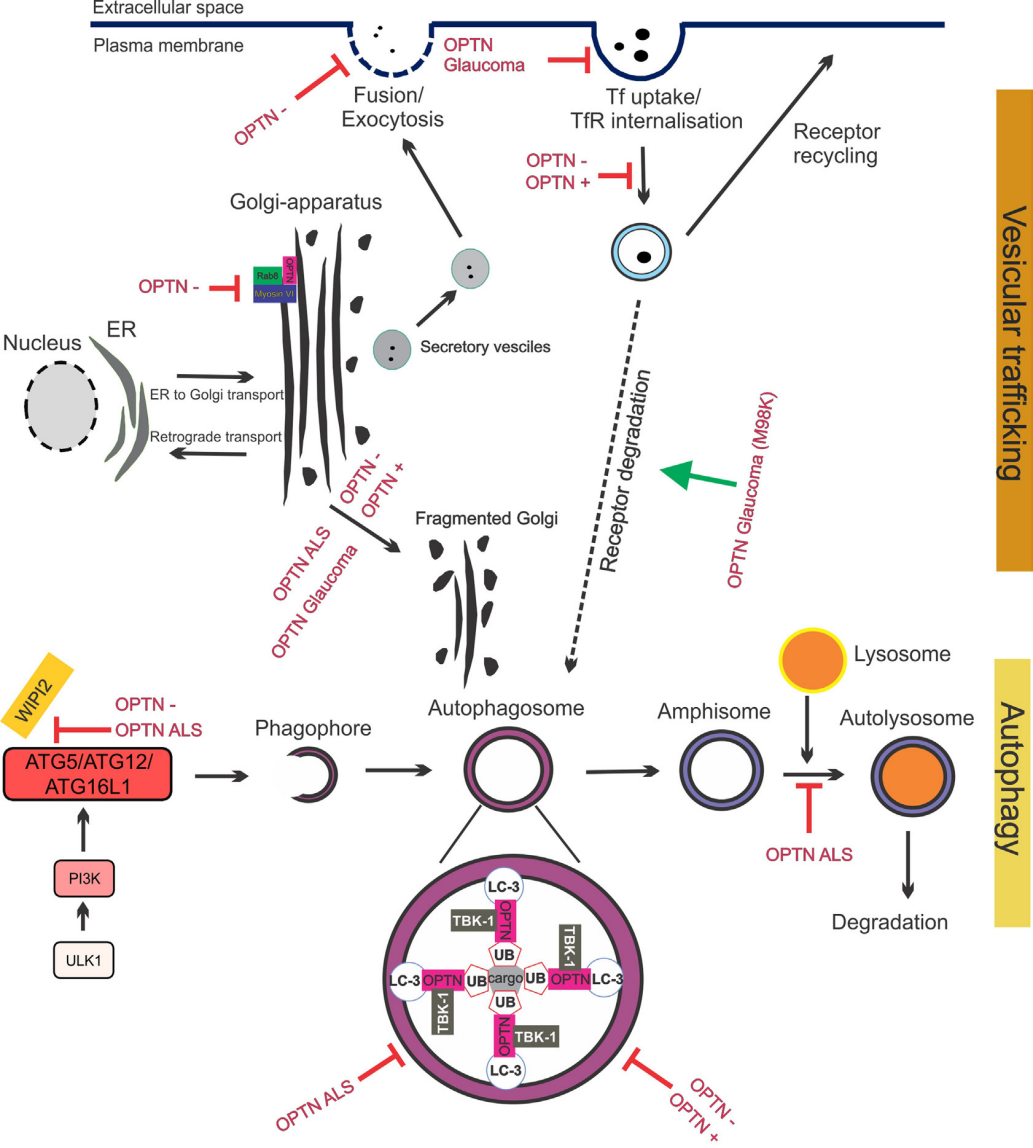
Optineurin mediates vesicular trafficking, receptor recycling and autophagy. (1) Optineurin controls Rab8 and myosin localization at the Golgi apparatus. Depletion of optineurin relocates the optineurin–Rab8–myosinVI complex from the Golgi. (2) Depletion of optineurin or overexpression of wild-type, ALS, or glaucoma mutant optineurin, results in Golgi fragmentation. (3) During vesicular trafficking, secretory vesicles travel from the Golgi to the plasma membrane where they fuse and undergo exocytosis. Depletion of optineurin abolishes the fusion of vesicles with the plasma membrane. (4) Transferrin receptor is recycled to the surface after transferrin uptake. E50K glaucoma mutant optineurin inhibits transferrin uptake, whilst depletion and overexpression of optineurin abolishes the recycling of transferrin receptor. M98K glaucoma mutant induces autophagic degradation of transferrin receptors by targeting them to autophagosomes. (5) Optineurin disrupts autophagy at several stages. The formation of the phagophore and autophagosome is inhibited by depletion of optineurin, as well as overexpression of ALS mutant optineurin, by inhibiting ATG5/ATG12/ATG6L1 complex formation. Optineurin links the ubiquitinated cargo to LC3, together facilitating autophagosomal engulfment of the cargo. Degradation of cargo, such as mitochondria and protein aggregates, is inhibited by both ALS and glaucoma mutant optineurin. Imbalance of optineurin expression, such as its overexpression or depletion, also interferes with autophagy, decreasing the clearance of cargo. ALS-associated mutant optineurin further blocks the fusion of the autophagosome to the lysosome, further inhibiting autophagy flux. Abbreviations: OPTN−: optineurin depletion, OPTN+: optineurin overexpression, OPTN ALS: ALS mutant optineurin, OPTN glaucoma: glaucoma mutant optineurin, Tf: transferrin, TfR: transferrin receptor, ER: endoplasmic reticulum.

The important role of optineurin in vesicular trafficking in complex with myosin VI and Rab8 is now well established (Figure 4). Myosins are motor proteins that move cargo along actin tracks (97). The Rab8-binding site is localized within the N-terminal domain of optineurin (60), whereas the myosin VI-binding region is located at its C-terminus (Figure 2). Optineurin co-localizes with both Rab8 and myosin VI at the Golgi apparatus and in vesicles in the cytoplasm (53, 98). The co-localization between myosin VI and Rab8 is optineurin dependent (98), suggesting that optineurin controls the assembly of the whole complex. This complex mediates Golgi organization (99), post-Golgi trafficking, exocytosis (100), and the basolateral delivery of membrane proteins (101).

In addition, optineurin mediates the binding between Rab8 and htt (60) and the latter is also thought to function in vesicular trafficking (60, 102). Htt interacts with the C-terminus of optineurin (Figure 2) and this interaction is dependent on the GTPase-activity of Rab8, as dominant-negative forms of Rab8 do not bind to optineurin (103). Furthermore, optineurin negatively controls Rab8 activity by interacting with its GTPase-activating protein (GAP) TBC1D17, thus perturbing Rab8-TBC1D17 interaction (103). Hence, optineurin provides the binding platform for myosin VI, Rab8, and htt, leading to their indirect interaction and regulation of vesicle trafficking. Hence together these studies establish an important regulatory function for optineurin in protein trafficking, via its interaction with key proteins implicated in vesicle formation and transport.

In most cell types, optineurin displays a vesicular, punctate pattern around the nucleus, Golgi, and plasma membrane (44, 53, 104–106). However, there is controversy as to how optineurin influences the Golgi apparatus and whether optineurin is a constant component of Golgi structure or is only transiently recruited there. Studies on perturbation of Golgi structure have suggested that optineurin plays a role in the function of the Golgi rather than in the maintenance of its structure (99). However, in a few cell types, such as retinal pigment epithelial cells (98) and normal rat kidney (NRK) cells (53), optineurin does not associate with the Golgi apparatus.

The Golgi normally undergoes extensive morphological changes during the cell cycle. Fragmented Golgi are produced during perturbed vesicular trafficking and in pathological conditions (107). Golgi fragmentation has been detected in cells overexpressing wild-type optineurin (98) and those depleted of optineurin (53, 54, 98), although this finding could not be reproduced in zebrafish (108). Depletion of optineurin from NRK cells results in loss of myosin VI from the Golgi and a disrupted Golgi ribbon structure (53). This implies that although optineurin is not located at the Golgi in NRK cells it still may influence Golgi function by directing myosin VI toward the Golgi. Nevertheless, this finding suggests that the levels of optineurin need to be tightly controlled to maintain Golgi morphology, suggesting a central role for optineurin in Golgi-mediated cellular processes. Whilst the absence of myosin VI inhibits ER-Golgi transport, depletion of optineurin had no effect on this process (100). However, there is evidence that optineurin functions in the post-Golgi part of the secretory pathway. Optineurin depletion affected vesicular fusion at the plasma membrane, which may involve other Rabs besides Rab8 (100).

There is also evidence that optineurin functions in the endocytic recycling of cell surface receptors. Depletion (55) or overexpression (56) of optineurin impairs endocytic trafficking of transferrin receptor (TfR) to the perinuclear region. Whilst optineurin did not co-localize with the early (Rab5 and Rab4) or late endosomal markers (Rab7) (56), a small portion co-localized with markers of recycling endosomes: TfR, Rab11 (55), and Rab8 (56). This appears to be mediated primarily by the UBD (55). These findings are consistent with those obtained from studies of classically secreted proteins. Depletion of optineurin led to reduced secretion of essential growth factors (neurotrophin 3 and ciliary neurotrophic factor) which induced apoptosis (54) and inhibited the delivery of epidermal growth factor receptor to the cell surface, leading to profound defects in lamellipodi formation. Again, depletion of myosin VI leads to similar defects, providing further evidence that optineurin and myosin VI possess a strong functional relationship (109). Hence together these data reveal that optineurin is important in secretory vesicle transport, endocytosis, and receptor recycling. Furthermore, recycling of cell surface receptors and efficient secretion of neurotransmitters are crucial for the conduction of action potentials at chemical synapses. Hence, dysfunction in these cellular processes could severely perturb important neuronal functions.

## The Role of Optineurin in Autophagy

Related to its function in vesicular trafficking, optineurin also has an important role in autophagy. Autophagy is a lysosomal degradation system that maintains intracellular homeostasis by degrading and recycling proteins/organelles. The most common type of autophagy is macroautophagy, which is characterized by the formation of the autophagosome, a double membrane vesicle that forms around the cargo. Autophagosomes fuse with endosomes, creating amphisomes, and the fusion of amphisomes or autophagosomes with the lysosome creates the autolysosome, where acidic degradation of cellular components takes place (110). In this review, we focus only on macroautophagy, referring to it as “autophagy” from here onward.

LC3 is a central protein in the autophagy pathway, which functions in the selection of autophagy substrates and autophagosome biogenesis. Autophagy begins with activation of the autophagy initiation complex, which consequently interacts with the phosphoinositide 3-kinase (PI3K) complex, leading to phagophore formation (Figure 4) (110). This is controlled by the ATG12:ATG5:ATG16L1 complex, which is recruited to the phagophore by WD repeat domain phosphoinositide-interacting protein 2 (Wipi2) (59). The ATG12:ATG5:ATG16L1 complex facilitates the lipidation of LC3 by conjugating phosphatidylethanolamine (PE). LC3-PE/LC3-II recruitment to the phagophore leads to elongation of the phagophore and closure of the double membrane, forming the autophagosome (110).

Based on cargo selection, two subtypes of autophagy can be distinguished; selective (111) and non-selective autophagy. Nonselective autophagy is the cellular stress response to nutrient deprivation, which targets non-essential molecules and organelles to the autophagosome to compensate for lack of nutrients in the environment. During selective autophagy, a specific cargo is selectively targeted, hence it may be considered as a housekeeping function. Specific subtypes of selective autophagy exist depending on the cargo targeted for degradation, including xenophagy (bacteria), mitophagy (mitochondria), and aggrephagy (protein aggregates). Neurons are post-mitotic; hence, they are particularly dependent on autophagy to dilute dysfunctional proteins and organelles. Thus, highly precise protein quality control systems are essential for the survival of neurons and their specialized morphologies require specific adaptations of autophagy, as well as other cellular processes (111, 112).

Optineurin is an autophagy receptor; hence, it tethers cargo for degradation to the autophagosome. Furthermore, multiple studies have indicated that optineurin is an important regulator of autophagy. Optineurin interacts with LC3 via its LC3 interacting motif (LIR, Figure 2), and the importance of this binding site is demonstrated by the fact that mutation to the LIR domain (F178A) abrogates LC3 binding to optineurin (49). Depletion of optineurin leads to significantly lower levels of LC3-II and fewer autophagosomes and autolysosomes, suggesting that optineurin is necessary for autophagy induction or autophagosome formation (59). Furthermore, optineurin-depleted cells display impairment of ATG12-ATG5-ATG16L1 complex recruitment to Wipi2, implying that optineurin influences autophagy before the involvement of Wipi2 (59). Hence optineurin may be necessary for the formation of the phagophore and hence autophagosome, although there is also evidence that optineurin is involved in autophagosome-lysosome fusion (113). Post-translational modifications of optineurin are also known to influence its function during autophagy. Ubiquitination of optineurin affects its interaction with other autophagy receptors and also targets it for degradation. HACE1 promotes K48-linked ubiquitination (47), which occurs predominantly at Lys193 of optineurin, which interestingly, is outside of the LC3-binding region. This in turn promotes the interaction of optineurin with autophagy receptor p62, leading to formation of the optineurin-p62 complex and acceleration of autophagy flux (47).

### The Role of Optineurin in Selective Autophagy

Optineurin is implicated in several different forms of selective autophagy including xenophagy, mitophagy, and aggrephagy. In this section, we focus on mitophagy and aggrephagy as the forms of selective autophagy that are the most relevant to neurodegenerative disorders.

### Role of Optineurin During Mitophagy

Mitochondria that need to be degraded are first ubiquitinated, and this is the signal for autophagic engulfment. Damaged mitochondria are recognized by PTEN-induced kinase 1 (PINK1), which subsequently recruits parkin, an E3 ubiquitin ligase (114). Parkin ubiquitinates mitochondria, and then ubiquitin is recognized by optineurin and other autophagy receptors, such as p62 and NDP-52, via their UBDs, which recruit the autophagic machinery. Optineurin was first shown to be recruited to damaged mitochondria in a parkin-dependent manner in HeLa cells and its depletion resulted in inhibition of LC3 recruitment to these mitochondria (115). Optineurin also recruits TBK-1 to poly-ubiquitinated mitochondria, where in turn, TBK-1 phosphorylates optineurin, enhancing its binding to ubiquitin and facilitating LC3 recruitment and mitophagy (46). Interestingly, optineurin and p62, which also function in mitophagy, localize to different parts of the mitochondria, independently of each other. p62 was not required for optineurin/LC3 recruitment to damaged mitochondria in HeLa cells, implying that optineurin and p62 regulate mitophagy by different mechanisms (115). Despite this, TBK-1 phosphorylates both optineurin and p62 during mitophagy (116). In another study, optineurin and two other autophagy receptors, NDP-52 and TAX1BP1, were recruited to depolarized mitochondria in HeLa cells but only depletion of optineurin blocked efficient mitophagy (57). This finding provides evidence for the selective involvement of specific autophagy receptors in mitophagy, indicating that optineurin has a central role in this process.

### Role of Optineurin in Aggrephagy

Protein inclusions are the pathological hallmark of neurodegenerative diseases and their presence implies dysfunction in protein degradation systems, either the UPS and/or autophagy. However, whilst the ubiquitin-proteasome system (UPS) degrades short-lived and soluble proteins, insoluble material, such as misfolded protein inclusions, are usually cleared by a subtype of selective autophagy, aggrephagy. Depletion of optineurin leads to increased aggregation of mutant G93C SOD1 and htt in HeLa cells, suggesting that it plays a role in aggrephagy (51). Similarly, overexpression of optineurin in Neuro2a cells prevented htt aggregation in an UBD-dependent manner (113). Interestingly, a relationship between optineurin and TBK-1 exists in aggrephagy and both proteins are mutated in fALS/sALS. Lack of TBK-1 expression and co-depletion of both optineurin and TBK-1 leads to the accumulation of protein aggregates to the same extent as depletion of optineurin alone, suggesting that optineurin function in aggrephagy is highly dependent on, and mediated by, TBK-1 (51).

## Involvement of Optineurin in Neurodegeneration in ALS and Other Neurodegenerative Diseases

Whilst optineurin has not been well studied in relation to neurodegeneration, optineurin dysfunction is associated with several cellular mechanisms that are implicated in the pathogenesis of ALS and glaucoma.

### The Role of Optineurin in Neuroinflammation in ALS

Increasing evidence suggests that neuroinflammation plays an important role in ALS pathogenesis. Overexpression of Toll-like receptor (TLR) 2/4 receptors in glial cells and TLR4 in motor neurons, as well as T-cell infiltration into the CNS (117, 118) and microglia activation, is observed in ALS patients (64). A recent study showed that the presence of regulatory T (Treg) cells slows down disease progression in SOD1 G93A transgenic mice. Furthermore, in ALS patients, there is an inverse correlation between the number of Treg cells and disease progression (117). However, the number of Treg cells decreases in rapidly progressing ALS patients (119). These findings highlight the importance of T cells in ALS progression. Importantly, microglia activation is an early event during ALS development, implying a role in disease pathogenesis (69, 120). In chimeric mice overexpressing wild-type and mSOD1 (G93A and G85R), non-neuronal cells that do not express mSOD1, including microglia, extend the survival of mSOD1 expressing motor neurons (121). Another study using the Cre/Lox system observed slower disease progression upon G37R SOD1 depletion in microglia (122). Furthermore, G93A SOD1 expressing microglia that are activated at the early stage of disease exhibit neuroprotective features that promote neuronal survival, whilst microglia from end stage animals display neurotoxic properties and induction of neuronal death (123). TLR4 antagonists are protective against the death of motor neurons co-cultured with microglia expressing mutant SOD1, and in a mouse model of spontaneous motor neuron degeneration, the wobbler mouse (124, 125). In addition, depletion of TLR4 prolongs the survival of G93A SOD1 mice (125). ATP release by dying motor neurons may activate microglia via ionotropic P2X and metabotropic P2Y purinergic receptors, leading to an inflammatory response. Interestingly, the levels of P2X increase in activated microglia in spinal cords of ALS patients and mutant G93A SOD1 transgenic mice (126, 127).

There is also evidence that neuroinflammation is involved in optineurin-associated ALS as well as in other forms of ALS. In sALS patients and fALS patients bearing optineurin mutations, NF-κB immunoreactivity in microglia is increased compared to controls and it is absent from the neuronal nucleus of patients (128). Depletion of optineurin increased NF-κB activity in neuronal cells leading to cell death, and wild-type overexpression of optineurin was able to rescue cell death, whilst ALS mutant optineurin could not (129) (Figure 3). Another study by Ito and colleagues demonstrated that in optineurin-depleted mice, severe axonopathy developed due to increased necroptosis (62). Furthermore, decompaction of myelin sheets due to oligodendrocyte death, and degeneration and swelling of motor neuron axons was observed. Interestingly, similar findings are observed in spinal cord of ALS patients (130). In the same study, optineurin depletion was carried out in specific cell types, including motor neurons, oligodendrocytes, astrocytes, and myeloid linages (62). This axonopathy and myelin abnormalities were only observed when optineurin was depleted from oligodendrocytes or microglia, and not from motor neurons or astrocytes, and it was linked to oligodendrocyte necroptosis. Interestingly, optineurin depletion in microglia only also led to axonopathy, suggesting that microglia contribute to oligodendrocyte health. Furthermore, depletion of optineurin did not result in motor neuron death in this model, even after 2 years. Whether depletion of optineurin in oligodendrocytes and microglia leads to a motor phenotype and ALS-like symptoms remains to be determined. Nonetheless, these data indicate that oligodendrocytes and microglia contribute to optineurin-mediated neurodegeneration.

### The Role of Optineurin in NF-κB Signaling in Glaucoma

There have been few studies examining the role of optineurin in NF-κB signaling in glaucoma, and these studies have yielded conflicting findings. Expression of the glaucoma mutant E50K in RGCs revealed no difference in interferon regulatory transcription factor 3 (IRF3) activation compared to cells expressing wild-type optineurin (131), suggesting that this pathway may not be implicated in glaucoma. However, expression of another glaucoma mutant, H486R, resulted in an altered interaction of optineurin with CYLD compared to wild-type, resulting in a lack of NF-κB inhibition, similar to studies in which optineurin was knocked out. In cells expressing the same mutant, CYLD displayed decreased binding to RIP and deubiquitination of RIP was decreased, suggesting that CYLD binding and deubiqutination of RIP are optineurin-mediated (63). Mutant H486R also fails to inhibit NF-κB activation upon IRAK1-mediated activation (61). These findings suggest that whilst the link between optineurin, the NF-κB pathway, and glaucoma is not well established, the NF-κB pathway is dysfunctional and highly activated in glaucoma.

### ER Stress and Secretory Defects in ALS and Glaucoma

Endoplasmic reticulum stress and fragmentation of the neuronal Golgi apparatus are now well-established cellular mechanisms associated with ALS (107, 132). The ER is the major site of protein folding, and if misfolded proteins accumulate, induction of the unfolded protein response occurs. Dysfunctional cellular trafficking is associated with ER stress and impaired cellular viability in all cell types, but neurons may be particularly susceptible to this type of stress, due to their extended and complex morphologies.

We have shown that expression of either wild-type or ALS-associated mutant optineurin (E478G and Q398X) induced ER stress in motor neuron-like cell lines (NSC-34), in contrast to the E50K glaucoma mutant, although this induction was significantly enhanced in the mutant expressing cells (52). Furthermore, this was accompanied by the inhibition of secretory protein transport from the Golgi to plasma membrane, Golgi fragmentation, and abnormal cytoplasmic distribution of optineurin in cells expressing the ALS-associated mutants. These findings were also associated with disruption of optineurin binding to myosin VI. Interestingly, optineurin binding to myosin VI was also decreased in spinal cord lysates from sALS patients compared to controls, implying that dysfunction to optineurin is also present in the much more common sporadic forms of ALS (52). Consistent with these findings, Golgi fragmentation was detected in 70% of anterior horn cells from an ALS patient bearing the E478G mutation (133) and also in another patient with the Q398X mutation, although optineurin immunoreactivity was absent from both the brain and spinal cord in the latter patient (134). Hence, together these data indicate that dysfunction to the ER and Golgi compartments are consistent features of ALS patients carrying optineurin mutations, as well as other forms of ALS.

Secretory defects have also been associated with optineurin in glaucoma. Expression of the E50K mutant reduced mir9 levels in RGC-5 cells, leading to increased levels of the transcriptional repressor RE1, and decreased levels of brain-derived neurotrophic factor (BDNF) (135). Hence, optineurin may mediate neural growth in RGCs through transcriptional control. The glaucoma mutant E50K forms larger and more granular structures compared to wild-type optineurin, inducing severe Golgi fragmentation (98). Furthermore, despite co-localizing with TfR, expression of E50K leads to significant inhibition of uptake of transferrin (Tf) (55, 56), indicating that whilst mutant optineurin can be recruited to TfR, uptake of Tf and recycling of TfR is abolished. E50K optineurin also leads to enhanced inhibition of Rab8 by TBC1D17, causing defective endocytic recycling of TfR (103) and linking this to RGC death. Furthermore, optineurin-positive vesicles displayed slower dynamics in E50K expressing cells compared to wild-type optineurin cells (55). Another glaucoma mutant, M98K, displayed enhanced interaction and co-localization with TfR, accompanied by decreased Tf uptake, similar to E50K optineurin (136). In contrast to E50K or wild-type optineurin overexpressing cells, the levels of TfR were lower in M98K overexpressing cells. This was due to increased autophagic degradation of TfR mediated by Rab12, leading to inhibition of cell death, which could be rescued by Rab12 depletion. Furthermore, M98K displays increased association with Rab12 compared to wild-type, suggesting that a toxic gain of function mechanism may exist (136).

Transgenic mice overexpressing E50K display a pronounced loss of RGCs and connecting synapses in the peripheral retina by 16 months of age, leading to thinning of the nerve fibre layer. This was associated with disrupted interaction between optineurin and Rab8 (137). Furthermore, in another mouse model, reactive gliosis was associated with deposition of mutant optineurin in the outer plexiform layer of the retina. In cell culture, E50K forms a stronger interaction with TBK-1 than with wild-type optineurin and this prohibited the self-oligomerization of E50K optineurin, inhibiting its intracellular degradation. In contrast, inhibition of TBK-1 abrogated the aberrant insolubility of E50K (138). Hence, together these data indicate that optineurin is involved in the pathogenesis of ALS and glaucoma. However, it remains unknown whether mutant forms of optineurin induce the same or distinct disease mechanisms in these disorders (Figure 1). It is tempting to speculate that the vesicular trafficking disturbances at least are shared mechanisms between both disorders, but further studies are required to state this conclusively.

### Optineurin-Related Autophagy Defects in ALS, Glaucoma, and Alzheimer’s Disease

Similarly, ALS-associated mutations in optineurin are known to induce dysfunction to autophagy. The E478G mutant displays impaired ability to form ATG16L1-positive puncta as well as phagophores and autophagosomes, and it does not form a complex with Atg12-5 and Wipi2 as readily (59). Optineurin mutants lacking the UBD do not co-localize with ATG12, ATG16L1 or Wipi2 puncta, in contrast to other mutants (59), highlighting the importance of the UBD in phagophore formation. Comparable results were observed in E478G and UBD-deleted optineurin overexpressing cells, where diminished autophagy induction was evident by fewer LC3 puncta upon serum starvation (113). Similarly, we have shown that in NSC-34 cells, expression of either ALS mutants E478G or Q398X, but not the glaucoma mutant E50K, led to inhibited autophagosome maturation into autolysosomes and accumulation of autophagosomes (52). During mitophagy, unlike wild-type optineurin, overexpression of ALS mutant E478G could not rescue inhibition of LC3 recruitment to mitochondria in optineurin-depleted cells. Furthermore, ALS mutants E478G or Q398X failed to relocate to mitochondria or induce mitophagy, unlike wild-type optineurin (57). As the E478G mutation is present within the UBD, this indicates that LC3 and ubiquitin binding are equally important to optineurin-mediated autophagy (115).

Optineurin co-localizes with mutant SOD1 (G93C) aggregates in HeLa cells (51) via its C-terminal coil-coiled domain and this interaction is independent of the UBD. These mSOD1 aggregates are positive for autophagosome markers LC3 and p62 as well as ubiquitin, suggesting an ongoing autophagic event (51) and indicating that optineurin is involved in the autophagic clearance of SOD1 aggregates. Interestingly, there is evidence that Aβ fibrils associated with Alzheimer’s disease are degraded by microglia via autophagy, or more specifically, aggrephagy, involving optineurin (139). Depletion of specific autophagy genes (*Atg7* and *Map1lc3B*) leads to increased levels of Aβ aggregates in microglia, without interfering with the uptake of Aβ fibrils. Furthermore, optineurin co-immunoprecipitates with Aβ fibrils, indicating that a direct or indirect interaction exists. Depletion of optineurin via siRNA from microglia also prevented Aβ fibril degradation (139).

Transgenic expression of the E50K mutant in mice leads to age-related loss of RGCs and elevated LC3-PE levels compared to mice expressing wild-type optineurin. Furthermore, in E50K transgenic mice, mitochondrial fragmentation, fewer mitochondria, and increased autophagosome formation, were detected in axons of the glial lamina, implying that death of these cells is associated with mitophagy in these animals. Alternatively, it is possible that the decreased numbers of mitochondria cannot support the significant energy needs of these neurons. Transport of mitochondria within the axon did not change in E50K mice, implying that the loss of mitochondria was due to increased mitophagy, and not redistribution of mitochondria (140).

In contrast, treatment with rapamycin, an inducer of autophagy via the mechanistic target of rapamycin (mTOR) pathway, rescues apoptosis in mouse RGCs expressing the glaucoma mutant E50K. It was suggested that this was due to the elimination of optineurin via autophagy (141). It should be recognized however that mTOR is linked to many cellular pathways as well as autophagy. In contrast, a more recent *in vitro* study involving wild-type and E50K provided evidence that optineurin is not degraded by autophagy (48). Optineurin can be poly-ubiquitinated at K63R and K48R by Hrd1, but this mediates the proteasomal degradation of optineurin rather than autophagosomal degradation. It remains unclear precisely how optineurin turnover is controlled, and whether autophagy or the UPS is the main quality control system (142).

## Optineurin and the Balance Between Cytotoxity and Cytoprotection

Excitotoxicity is a pathological process by which neurons degenerate and die by excessive stimulation of receptors, due to binding of the excitatory neurotransmitter glutamate. These receptors include the metabotropic glutamate receptors (mGluRs) and the ionotropic AMPA and NMDA receptors (143). Optineurin functions as a negative regulator of mGlutR1a signaling by interacting with the mGluR1a receptor and attenuating its ability to couple to phospholipidase Cβ (144), which in turn decreases inositol phosphatase production and protein kinase C activation. Interestingly, htt with expanded glutamine regions (htt-G138) facilitates optineurin-mediated attenuation of mGluR1a signaling (144). However, whether optineurin-mediated inhibition of mGluR1a signaling results in beneficial or harmful consequences remains to be elucidated.

Interestingly, wild-type optineurin may exert protective effects on cellular survival. Overexpression of optineurin attenuates TNFα-induced cell death in HeLa cells (145) and ROS-mediated H_2_O_2_-induced cell death in NIH3T3 cells (105), although no protection was observed in RGC-5 cells (105, 145). This protection correlates with changes in the subcellular localization of optineurin, which translocates in a Rab8 GTPase-dependent manner from the Golgi apparatus to the nucleus. This translocation also occurs during cell division, and it is initiated by phosphorylation of optineurin by polo-like kinase 1 (Plk1) (146), which plays a pivotal role in cell-cycle progression through mitotic entry to cytokinesis (147). Depletion of optineurin increases Plk1 activity, which induces abscission failure and multi-nucleation of cells (146).

However, in some instances, wild-type optineurin can also induce toxicity, which is dependent on its ability to bind to Rab8 (148). In yeast, overexpression of wild-type optineurin-induced toxicity by a mitogen-activated protein kinase 25 (MAP2K5) kinase-dependent mechanism (148). This finding was confirmed in mammalian cells, where the formation of wild-type optineurin aggregates was reduced by treatment with a MAP2K5 inhibitor (148). The MAP2K/MAPK pathways are associated with both neuronal cell death (149) and survival (150). Whether MAP2K5-mediated cell death or survival is fine-tuned by optineurin remains to be elucidated. However, depletion of optineurin leads to inhibited cell growth in RGCs (151) and microarray analysis identified 112 genes that were differentially expressed in these cells compared to control cells expressing optineurin, including BDNF, neurotrophin 3, synaptosomal-associated protein 25 (SNAP25) and neurofilament light polypeptide. The lack of growth factors was associated with decreased growth in optineurin-depleted cells (151).

The ALS optineurin mutant E478G induced cell death specifically in motor neuron-like cells, compared to glaucoma mutants E50K or M98K, which did not affect cell survival. Furthermore, in contrast, the ALS mutants did not induce cell death in a photoreceptor cell line, 661W, whilst the glaucoma mutants did (152). Importantly, these data imply that cell death is induced by optineurin mutations only in the appropriate, specific cell type associated with each different neurodegenerative disease. Consistent with this notion, in an earlier study, M98K induced cell death in RGCs, but not in other neuronal or non-neuronal cells (IMR32, HeLa, and COS-1). Similarly, another glaucoma mutant, E50K, selectively induced ROS-associated-specific death of RGCs but not COS-1, HeLa or IMR32 cell lines, whilst overexpression of wild-type optineurin did not (136). However, other glaucoma-associated mutants (R545Q, H26D, and H486R) did not induce cell death, implying that E50K is more toxic than the other mutants (145, 153).

Overexpression of either ALS (E478G) or glaucoma optineurin mutants (E50K) resulted in a motor neuron axonopathy in zebrafish embryos, thus linking optineurin mutations to disease relevant phenotypes *in vivo*. Interestingly, knockout of MAPK2 rescued optineurin induced motor axonopathy in zebrafish (154), and the formation of E50K and E478G aggregates was inhibited by treatment with a MAP2K5 inhibitor in the absence of changes in expression level, suggesting that MAP2K5 has a direct effect on the formation of optineurin aggregates (154). Both wild-type and mutant E50K optineurin forms toxic, non-amyloid aggregates in yeast, and not surprisingly, E50K is more toxic than wild-type (148). This may be due to increased oligomerization of optineurin due to its enhanced binding to TBK-1 (82). Interestingly, wild-type and ALS mutant (E478G) optineurin display unique aggregation patterns *in vitro* compared to other aggregation prone proteins associated with ALS, including TDP-43 and FUS. Wild-type and E478G optineurin overexpression resulted in the formation of a single large inclusion in the majority of yeast cells, in contrast to cells expressing TDP-43 or FUS, that formed inclusions of multiple foci with heterogeneous size (148). This indicates that optineurin is an aggregation prone protein, consistent with the presence of inclusions.

## Optineurin Pathology in Neurodegenerative Diseases

Whilst neurodegenerative diseases display a diverse range of symptoms and affect different subtypes of neurons, a common phenomenon is the presence of protein aggregates. Interestingly, optineurin is present and localized with in the aggregates in a wide range of neurodegenerative diseases. Almost all (97%) ALS patients display TDP-43-positive protein inclusions in degenerating motor neurons (155), and in some patients, SOD1, FUS, and C9orf72 aggregates may also be present. TDP-43 pathology (ubiquitinated, hyper-phosphorylated, and truncated TDP-43) is a feature of FTD also, and it is present in 45% of patients (155). Another 45% of FTD patients present with inclusions containing misfolded microtubule stabilizing protein tau without TDP-43 pathology (156).

One study identified optineurin-positive inclusions in both fALS and sALS patients that co-localized with TDP-43 and ubiquitin (2). Similarly, in fALS patients bearing mutations in SOD1, SOD1 inclusions were also immuno positive for optineurin (2). In contrast, however, another study examining 32 sALS cases observed that the majority of TDP-43 inclusions were negative for optineurin (3). Furthermore, whilst 33% of FTD-TDP cases examined displayed cytoplasmic optineurin neuronal inclusions in the brain (3), the majority of TDP-43 inclusions did not co-localize with optineurin (3). Similarly, in another study, the inclusions present in a patient bearing the ALS E478G mutation were positive for ubiquitin, p62, and TDP-43, but they were not immunoreactive with optineurin (133). Furthermore, optineurin-positive inclusions were absent from FTD patients manifesting with taupathies and α-synucleinopathies (3). In a recently published study (157), a fALS patient bearing an optineurin mutation (E478G) displayed features of several proteinopathies, including phosphorylated TDP-43, phosphorylated tau, and α-synuclein, although it was not examined whether optineurin is present in these inclusions. Hence together these data indicate that the involvement of optineurin in TDP-43 pathology in ALS and FTD remains controversial.

As well as ALS and FTD, optineurin pathology has also been detected in other neurodegenerative disorders. Optineurin-positive inclusions in neuronal and glial cells are occasionally observed in Alzheimer’s disease; however, they do not co-localize with tau or TDP-43 (158). Furthermore, the optineurin immunoreactivity displays a different pattern compared to phospho-TDP-43, providing further evidence that TDP-43 and optineurin pathology are independent of each other. Marinesco bodies are small, spherical intranuclear inclusions that are observed in neurons of the substantia nigra and locus coerelus in Alzheimer’s disease, Parkinson’s disease, and dementia with Lewy bodies (159). These bodies co-localize and display strong immunostaining for optineurin in Alzheimer’s disease (4). Furthermore, optineurin co-localizes with htt and frequently occurs in the neuronal intracellular inclusions in Huntington’s disease and, to a lesser extent, in inclusions of the neuropil and perikaryon. These inclusions are common in interneurons and are present but less frequent, in medium projection neurons of the cortex and striatum (5). Similarly, in dominant hereditary motor and sensory axonopathy patients, autopsy samples displayed optineurin-positive inclusions (158). In postmortem tissue from sporadic basophilic inclusion body disease (BIBD) and fALS with FUS mutations, all basophilic inclusions examined were positive and co-localized with optineurin and FUS, but not TDP-43 or SOD1 (160). In contrast, optineurin-positive inclusions were co-localized with TDP-43 in muscle tissue of sporadic inclusion body myositis (sIBM) patients (161). Hence, similar to ALS/FTD, the involvement of optineurin in the pathology of other neurodegenerative disorders remains unclear. Whether optineurin is misfolded and actively contributes to the formation of these aggregates and directly causes cell death, or alternatively, is passively recruited to the inclusions via interaction with aggregation prone proteins, is still under investigation. An intriguing alternative scenario is that optineurin is recruited to the inclusions to direct them towards the autophagic pathway. Nevertheless, these data indicate that optineurin is part of the pathology of a wide range of neurodegenerative diseases, highlighting a central role for optineurin in protein aggregation.

## Conclusion

Dysfunctional optineurin is implicated in several neurodegenerative diseases, particularly ALS and glaucoma. In addition, the normal cellular functions of optineurin in neuroinflammation, autophagy, and vesicular trafficking, are important mechanisms that dysfunction in these disorders. Furthermore, mutations identified in familial forms of these disorders known to lead to aberrant autophagy, receptor recycling, and Golgi fragmentation, as well as unbalanced NF-κB signaling. However, these mechanisms are poorly defined and the relationship between optineurin and neurodegeneration has not been well studied in comparison to other proteins linked to ALS/glaucoma. Optineurin also has many putative binding partners. One possible scenario in neurodegenerative diseases is that these binding partners interact aberrantly with mutant optineurin. Previous studies have implicated a loss-of-function mechanism by reduced interaction with these proteins. However, it is also possible that mutant optineurin acquires additional, non-physiological binding partners in ALS, and/or glaucoma, resulting in a toxic gain-of-function mechanism. Further understanding of optineurin interactions is therefore needed to establish a conclusive link between neurodegeneration and altered interactions between optineurin and other proteins.

## Author Contributions

Both authors contributed to this article. JDA and RPT co-wrote and edited the article throughout for content and style consistency. RPT prepared the tables and figures.

## Conflict of Interest Statement

The authors declare that the research was conducted in the absence of any commercial or financial relationships that could be construed as a potential conflict of interest.
